# Periodontitis was associated with mesial concavity of the maxillary first premolar: a cross-sectional study

**DOI:** 10.1038/s41598-024-53371-y

**Published:** 2024-02-05

**Authors:** Feng Chen, Qi Liu, Xinyue Liu, Qian Fang, Bingxin Zhou, Ru Li, Zhe Shen, Kai Xin Zheng, Cheng Ding, Liangjun Zhong

**Affiliations:** 1 Stomatology Center, Affiliated Hospital of Hangzhou Normal University, Hangzhou Normal University, Hangzhou, 310015 China; 2 School of Stomatology, Hangzhou Normal University, Hangzhou, 310015 China

**Keywords:** Anatomy, Diseases, Health care, Medical research

## Abstract

The association between the anatomical features of teeth and the pathogenesis of periodontitis is well-documented. This study aimed to evaluate the influence of the mesial concavity of the maxillary first premolar on periodontal clinical indices and alveolar bone resorption rates. Employing a cross-sectional design, in 226 patients with periodontitis, we used cone beam computed tomography(CBCT) to examine the mesial concavity and alveolar bone resorption of 343 maxillary first premolar. Periodontal clinical indicators recorded by periodontal probing in the mesial of the maxillary first premolar in patients with periodontitis. Our findings indicate that the presence of mesial concavity at the cemento-enamel junction of the maxillary first premolar was not significantly influenced by either tooth position or patient sex (*p* > 0.05). Nonetheless, the mesial concavity at the cemento-enamel junction of the maxillary first premolar was found to exacerbate alveolar bone resorption and the inflammatory condition (*p* < 0.05). We infer that the mesial concavity at the cemento-enamel junction of the maxillary first premolar may contribute to localized alveolar bone loss and accelerate the progression of periodontal disease.

## Introduction

Periodontitis is an inflammatory disease that leads to the destruction of alveolar bone and periodontal ligaments, with dental plaque identified as the primary etiological agent^1^. Initial periodontal therapy typically involves the mechanical debridement of bacterial plaque from tooth surfaces, which includes systematic scaling and root planing (SRP), a procedure demonstrated to be effective^2–4^. However, studies report that post-SRP, the residual rate of dental calculus is approximately 57% when the periodontal probing depth (PD) ranges from 4 to 6 mm, escalating to 68% when PD exceeds 6 mm^5–7^. This persistence of plaque is often attributed to the unique anatomical complexities of teeth, including features such as root bifurcations, concavities, and grooves. These anatomical nuances present significant challenges to achieving thorough debridement, thereby playing a contributory role in the onset and progression of periodontal diseases^8^. Consequently, a comprehensive understanding of dental anatomy is imperative, as it aids periodontists in the effective removal of plaque.

The anatomical intricacies of the maxillary first premolar set it apart from other molars due to its varied morphology, which complicates periodontal treatment and poses a challenge for patient home care. A notable feature of the maxillary first premolar in individuals with periodontitis is the mesial concavity^9,10^. Ong and Neo categorized this concavity into five classifications based on location and observed that concavities originating at the enamel or the cemento-enamel junction foster plaque accumulation, thereby exacerbating periodontitis^11^. In research involving 107 extracted teeth, Knut et al. found a significant association between the extent of periodontal attachment loss and the presence of mesial concavity; teeth with root concavities exhibited more severe attachment loss compared to those without ^12^.

Although mesial concavity can be visualized and precisely measured post-extraction, the availability of first premolars is often limited due to loss primarily from orthodontic treatments or periodontitis, resulting in a sample size that lacks broader representation. This limitation has historically impeded research in this area. Cone-beam computed tomography (CBCT) effectively compensates for this shortfall. CBCT provides clear images across axial, sagittal, and coronal planes, eliminating structural superimposition and enhancing visualization of the target anatomy ^13^. Compared to two-dimensional imaging techniques, CBCT delineates tooth surfaces with greater clarity, enabling reliable and precise evaluations of the alveolar bone ^14^, thereby enriching our understanding of periodontitis^15^. Consequently, CBCT is employed in various dental applications, offering not only high-resolution depiction of dental anatomy but also dependable assessments of alveolar bone defect resorption^16^.

Previous investigations have established that mesial concavity influences the outcome of endodontic therapy and plays a pivotal role in dental restorations^11,17,18^, However, its association with periodontal tissues has seldom been explored. To bridge this gap, we assessed the mesial concavities of 343 maxillary first premolars in 226 periodontitis patients, examining the influence of these anatomical features on periodontal clinical parameters and bone loss through CBCT analysis. This study aims to offer novel insights into the development of periodontal disease.

## Results

### Distribution of maxillary first premolar mesial concavity in positions and sex

In this study, 343 maxillary first premolar teeth were analyzed, comprising 167 from the right side and 176 from the left. Of the right maxillary first premolars, 68.9% (n = 115) exhibited mesial concavity, while on the left, the prevalence was 62.5% (n = 110). The mean angles of mesial concavity for the right and left maxillary first premolars were 150.5 ± 9.59° and 152.3 ± 9.06°, respectively. No significant statistical differences were observed in the incidence or angle of mesial concavity between the right and left maxillary first premolars (*p* > 0.05).

Sex-based variation in the distribution of maxillary first premolar mesial concavity was also explored. The sample consisted of teeth from 177 females and 166 males. mesial concavity was present in 62.9% (n = 115) of male and 62.1% (n = 110) of female patients. Further analysis of the mean angle of mesial concavity by sex revealed angles of 150.3° for males and 152.5° for females, with these differences not reaching statistical significance (*p* > 0.05). The pertinent data is summarized in Table 1.

**Table 1:** Incidence of proximal mesial concavity of maxillary first premolar in positions and sex.

	Group	Number	Number of occurrences	Percentage (%)	The mean angle (Mean ± SD)
Position	Left	176	110	62.5	152.3 ± 9.06
	Right	167	115	68.9	150.5 ± 9.59
	*p*-value	–	–	0.21	0.13
sexs	Male	166	115	69.2	150.3 ± 10.24
	Female	177	110	62.1	152.5 ± 8.35
	*p*-value	–	–	0.22	0.17

### Effects of maxillary first premolars with mesial concavity on CAL

Clinical attachment loss (CAL) measurements for the maxillary first premolar were taken prior to initiating basic periodontal treatment. In patients with mesial concavity, the mean CAL was 3.48 ± 1.56 mm on the buccal side and 3.29 ± 1.38 mm on the palatal side. Conversely, in patients lacking mesial concavity, the mean CAL was 3.06 ± 0.91 mm on the buccal aspect and 3.22 ± 1.22 mm on the palatal aspect. The data revealed that the mean buccal CAL was greater in premolars with mesial concavity compared to those without, although this difference was not observed on the palatal side. Notably, the difference in buccal CAL associated with mesial concavity was statistically significant (*p* < 0.05). These findings are detailed in Table 2.

**Table 2:** Clinical attachment loss (CAL) of the first premolars without/with concavity (Mean ± SD).

Clinical attachment loss (CAL)	Dental with concavity	Dental without concavity	*p*-value
Buccal	3.48 ± 1.56	3.06 ± 0.91	0.02
Palatal	3.29 ± 1.38	3.22 ± 1.22	0.40

### Affected the PI, BOP, and GI by mesial concavity

Mesial Plaque Index (PI) values were recorded for patients who had not received basic periodontal treatment. Among periodontitis patients with mesial concavity, 11.1% exhibited a PI score of 0, 38.2% had a score of 1, and 41.8% had a score of 2. In contrast, for periodontitis patients without mesial concavity, the scores were distributed as follows: 25.4% had a PI score of 0, 38.1% had a score of 1, and 28.0% had a score of 2. Specifically, PI scores of 0 on the mesial aspect of the first premolar with concavity were 9%, compared to 8% for those without concavity. The influence of mesial concavity on PI was statistically significant (*p* < 0.05). These results are detailed in Table 3.

**Table 3:** Plaque index of the maxillary first premolars without/with mesial concavity.

	The plaque index was 0	The plaque index was 1	The plaque index was 2	The plaque index was 3	*p*-value
With mesial concavity	25 (11.1%)	86 (38.2%)	94 (41.8%)	20 (9%)	0.03
Without mesial concavity	30 (25.4%)	45 (38.1%)	33 (28.0%)	10 (8%)	

Bleeding on Probing (BOP) was also assessed prior to basic periodontal treatment. The percentage of bleeding upon probing the mesial buccal aspect of the maxillary first premolar with concavity was 49.3%, compared to 37.2% when the concavity was absent. The impact of mesial concavity on BOP was statistically significant (*p* < 0.05). However, when probing the mesial palatal aspect, BOP was recorded at 43.5% for premolars with concavity and 35.5% for those without concavity. In this case, mesial concavity did not significantly affect the rate of palatal bleeding (p > 0.05). These findings are presented in Table 4.

**Table 4:** Effect of mesial concavity on palatal and buccal percentage of bleeding on probing.

	Palatal			Buccal		
	Bleeding on probing	No bleeding on probing	Percentage (%)	Bleeding on probing	No bleeding on probing	Percentage (%)
Dental with concavity	98	127	43.5	111	114	49.3
Dental without concavity	42	76	35.5	44	74	37.2
*p*-value	–	–	0.15	–	–	0.03

Similarly, at the patient's initial visit, the gingival index (GI) of the patient's mesial maxillary first premolar was observed and the gingival index was recorded. Within the periodontitis group suffering from mesial concavity, 20.4% exhibited a GI index of 0, 36.0% a GI index of 1, 29.8% a GI index of 2, and 13.8% a GI index of 3. However, for periodontitis patients without mesial concavity, the scores were distributed as follows:22.0% had a PI score of 0, 49.2% had a score of 1, 17.8% had a score of 2, and 11.0% a GI index of 3. The statistical results showed that GI was higher in patients with mesial concavity(*p* < 0.05). The specific data are shown in Table 5.

**Table 5:** Gingival index of the maxillary first premolars without/with mesial concavity.

	The gingival Index was 0	The gingival Index was 1	The gingival Index was 2	The gingival Index was 3	*p*-value
With mesial concavity	46 (20.4%)	81 (36.0%)	67 (29.8%)	31 (13.8%)	0.04
Without mesial concavity	31 (22.0%)	54 (49.2%)	21 (17.8%)	12 (11.0%)	

### Impact of mesial concavity on alveolar bone resorption

In patients with periodontitis, cone-beam computed tomography (CBCT) images provide precise measurements of alveolar bone height, facilitating the assessment of alveolar bone loss. Our analysis indicates that mesial concavity exacerbates alveolar bone resorption on the buccal aspect of the first premolar. Across all age groups, the difference in the rate of buccal alveolar bone loss associated with concavity was statistically significant (*p* < 0.05), while the rate of palatal alveolar bone resorption was not significantly affected (*p* > 0.05). These findings are illustrated in Table 6.

**Table 6:** Impact of mesial concavity on buccal and palatal alveolar bone absorbs.

Age group (years)	The buccal alveolar bone absorbs (Mean ± SD%)			The palatal alveolar bone absorbs(Mean ± SD%)		
	Dental with	Dental without concavity	*p*-value	Dental with concavity	Dental without concavity	*p*-value
≤ 30	8.63 ± 7.45	5.54 ± 5.50	0.04	5.70 ± 9.09	5.80 ± 6.35	0.46
31–40	15.1 ± 13.62	8.58 ± 6.10	0.03	11.97 ± 14.07	7.58 ± 5.91	0.57
41–50	18.29 ± 10.33	11.48 ± 7.92	0.04	12.65 ± 10.53	13.10 ± 10.69	0.61
≥ 51	23.06 ± 12.11	15.34 ± 7.62	0.01	19.18 ± 15.01	15.14 ± 7.68	0.76

### The severity of periodontitis in the maxillary first premolars with the degree of mesial concavity

Using clinical attachment loss as a criterion, we graded 225 maxillary first premolar teeth: clinical attachment loss of 1–2 mm was classified as mild periodontitis, clinical attachment loss of 3–4 mm as moderate periodontitis, and clinical attachment loss of more than 5 mm as severe periodontitis^19^. For teeth with mild periodontitis, the angle of mesial concavity was measured at 154.3 ± 6.2; for teeth with moderate periodontitis, it was 150.5 ± 9.5; and for teeth with severe periodontitis, it was 149.9 ± 10.1. We contrasted all of the groups. We discovered significant differences between the mild and moderate periodontitis groups and the mild and severe periodontitis groups (*p* < 0.05), but in the comparison of the moderate and severe periodontitis groups, no significant difference was found (*p* > 0.05). The specific data are shown in Table 7.

**Table 7:** The severity of periodontitis in the maxillary first premolars with the degree of mesial concavity (Mean ± SD).

Group	Number	The mean Angle of mesial concavity		*p*-value
Mild periodontitis	64	154.3 ± 6.2	Mild periodontitis	0.02
			Moderate periodontitis	
Moderate periodontitis	111	150.5 ± 9.5	Mild periodontitis	0.04
			Severe periodontitis	
Severe periodontitis	50	149.9 ± 10.1	Moderate periodontitis	1
			Severe periodontitis	

## Discussion

In the present study, we investigated the prevalence of mesial concavity at the cemento-enamel junction in patients with periodontitis and examined its impact on the disease. Our findings indicate a high prevalence of mesial concavity among patients with periodontitis, with no statistically significant differences in distribution based on tooth position or sex. Moreover, we demonstrated that mesial concavity contributes to increased inflammation and alveolar bone resorption in these patients, thus exacerbating periodontal disease, which is an advancement over previous research.

Mesial concavity, also recognized as furcal or developmental concavity, is commonly observed at the cemento-enamel junction^20^. Prior studies have reported a wide range of prevalence for this anatomical feature in maxillary first premolars, from 62 to 100%^21,22^. In the North American population, Fox et al. found a 100% occurrence in the North American population^23^. However, our study identified a prevalence rate of 65.6%, which may be lower due to our specific focus on the cemento-enamel junction and could also reflect geographic and ethnic variation in the anatomy of the maxillary first premolar^23^. Our results regarding the absence of sex and positional differences in the occurrence of mesial concavity are consistent with previous literature^11^. In a novel approach, our study also analyzed the angle of mesial concavity using CBCT imaging in patients with periodontitis, enhancing our comprehension of the concavity's depth and offering valuable insights for clinical diagnosis and treatment planning.

Food particles and bacterial plaque are prone to accumulate in mesial concavities, facilitating calculus formation^24^. This anatomical feature complicates patients’ oral hygiene maintenance, with dental floss often proving ineffective for cleaning these concavities ^25^. Our study corroborates this by demonstrating a greater plaque index in the mesial depressions, with the concave buccal side also exhibiting higher rates of BOP, indicative of a more severe inflammatory state. The arrangement of teeth can further influence oral hygiene efficiency^26^; in the case of the maxillary first premolar, the thinner buccal and palatal cusps provide increased exposure of the palatal neighboring surfaces, potentially easing cleaning efforts on the palatal side.

CAL serves as a critical metric for gauging periodontitis severity^6^. Except for the groups with moderate and severe periodontitis, where no significant differences were observed, most likely as a result of sample size variations, there were statistically significant differences between the other groups. Our research revealed that maxillary first premolars with a more severe clinical attachment loss had a greater degree of mesial concavity. As a result, we may infer that mesial concavity is one of the factors that accelerates the development of periodontal disease. Mesial concavity allows teeth to better withstand torque and increases the attachment surface area, but it also promotes the retention of plaque and calculus, especially after attachment loss^27^. Scaling and root planing (SRP) is the cornerstone treatment for chronic periodontitis ^28^. Research has shown that concavities, a common morphological feature in maxillary premolars, may hinder periodontal therapy and complicate plaque control by clinicians^29^. It has been noted that debridement of calculus from periodontal pockets deeper than three millimeters poses a greater challenge, with difficulty further intensified when addressing teeth with concave root surfaces^30^. Periodontists should take the presence of concavity into account when devising early-stage periodontal treatment strategies. Deeper root surface concavities could be filled or otherwise restored to enable more comprehensive treatment of the root surface^29^.

Periodontal disease can lead to the resorption of alveolar bone, manifesting various alterations in bone architecture^31^. In our study, we stratified the sample by age to minimize experimental bias owing to the significant impact of age on alveolar bone resorption^32^. CBCT enables relatively precise measurements of alveolar bone height ^33^. The method we employed involved fixing points and drawing connecting lines to measure the bone, thereby reducing human error given that each point was distinct. Our findings indicate that mesial concavity affects the rate of buccal alveolar bone resorption more severely than on the palatal side, leading to asymmetric bone development. The presence of a mesial concavity in the maxillary first premolar may cause irregular morphologies of mesial alveolar bone defects^34^, corroborating our results. These anomalies in bone structure could facilitate plaque accumulation and impede its removal by periodontal professionals.

There are limitations in this study that warrant consideration. Firstly, while we relied on CAL, BOP, PI, and GI as markers of gingival inflammation and periodontal health, potential biases from our sample size—which was not large and was ethnically homogeneous—could not be overlooked. Our investigation focused solely on the impact of mesial concavity on periodontal health of the maxillary first premolar, without delving into the underlying mechanisms. Future research will address whether the microbial profile associated with concave maxillary first premolars differs and whether it influences inflammation levels in the mesial area.

In conclusion, our study establishes a connection between the mesial concavity of the maxillary first premolar and periodontitis. Periodontists should give special attention to this anatomical feature during routine treatment to enhance plaque removal, as mesial concavity is implicated in clinical attachment loss and alveolar bone resorption. Consequently, a thorough understanding of root anatomy is advocated, as it is a crucial prognostic factor in periodontal disease management.

## Methods

### Sample size calculation

There was a 0.7 CAL difference with a 0.3 standard deviation between patients with and without mesial concavity, according to the literature^11^. Ninety patients were included in the study based on how the statistical sample size was assessed, with a two-sided test with a power of 90% and a test level alpha of 0.05, accounting for the possibility that the sample was lost to follow-up. Taking into account that the sample may have been lost to follow-up, the number of patients included in the study was 90 based on how the statistical sample size was estimated, with a test level alpha of 0.05 and a two-sided test with a power of 90%.To make the experimental data more credible, We recruited 226 patients and included 343 maxillary first premolar teeth.

### Recruitment of research participants

**Figure 1:**
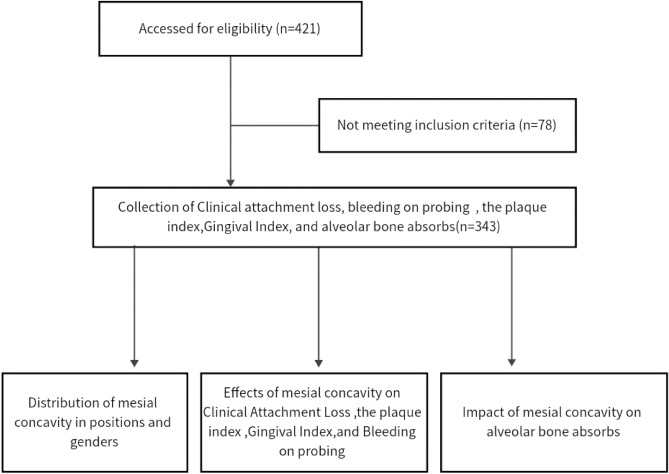
Flow chart of the study design.

This study was conducted in strict adherence to the protocols of a cross-sectional study and met all operational specifications. Informed consent was obtained from all participating patients. The research received ethical approval from the ethics committee of the affiliated hospital of Hangzhou Normal University (Approval No. 2022(E2)-KS-107). Patients who underwent CBCT at the Center for Stomatology, Affiliated Hospital of Hangzhou Normal University, between November 2021 and March 2023 were considered for inclusion. Specific criteria for inclusion were: (1) diagnosis of periodontitis, (2) age between 18 and 80 years, (3) absence of systemic diseases such as diabetes and hyperthyroidism, or other conditions affecting bone metabolism, and (4) at least one fully developed maxillary first premolar. From an initial total of 421 teeth, exclusions were made for 4 teeth with root resorption, 5 with root hypoplasia, 18 with inadequate imaging for evaluation, 23 with a history of root canal treatment leading to root contour alteration, and 28 deemed non-restorable. Consequently, 343 first premolars from 266 patients were ultimately included in the study (Fig. 1).

### Acquisition of clinical data

**Figure 2:**
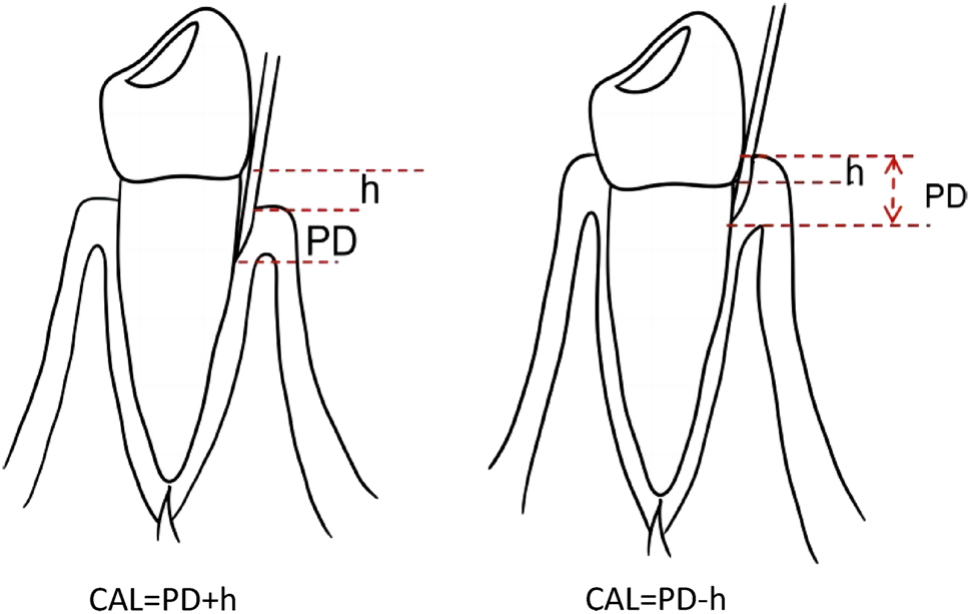
CAL (clinical attachment loss); PD = (probing depth); h = gingival margin level.

Periodontal clinical data were collected by an experienced periodontist. The periodontal status of the maxillary first premolar was assessed at six sites using a periodontal probe (Hu-Friedy; PCPUNC156, America), and clinical parameters such as PD, CAL, BOP, PI, and GI were recorded. A consistent force of 20 g was applied to measure PD from the gingival margin in millimeters on the mesial buccal and palatal sides of the maxillary first premolar. CAL was calculated as the sum of PD and the gingival margin level in cases of gingival recession(h) or as the difference between PD and the gingival margin level(h) when the gingival margin covered the CEJ (Fig. 2). Mesial PI was scored from 0 to 3^35^, with 0 indicating no visible plaque at the gingival margin, 1 assigned for thin plaque detectable with probing but not visible to the naked eye, 2 for moderate plaque deposits visible without magnification, and 3 for abundant plaque accumulation on the gingival margin and adjacent surfaces. Mesial GI was scored from 0 to 3^36^, 0 = normal gums; 1 = moderate inflammation: mild color change and mild edema, no bleeding on probing; 2 = Moderate inflammation: red gums, shiny edema, bleeding on probing; 3 = Severe inflammation: gums are visibly red, swollen or ulcerated, with a tendency to bleed spontaneously. Reliability of measurements was ensured with kappa coefficients of 0.82 for CAL,0.86 for PI,0.84 for GI, and 0.84 for Percentage of bleeding on probing.

### Image acquisition

Images were captured using a CBCT scanner (Galileos, Sirona, Germany) equipped with a proven hybrid 3D solution, featuring an optimal 8 × 8 cm cylindrical volume at a resolution of 160 µm. A Sirona Dental System (D-64625 Bensheim, Germany) was used, operating at 85 kVp and 7 mA. The images were acquired and displayed on a 17-in. personal computer (PC) monitor, allowing for the visualization of transverse slices in the axial, coronal, and sagittal planes. Image reconstruction was processed using the GALAXIS 1.9 software (SICAT GmbH & Co. KG, Bonn, Germany).

### Image measurements

The full texts of all potentially eligible articles were independently reviewed by two authors (Xinyue Liu and Feng Chen). Data from studies meeting the inclusion criteria were extracted and tabulated using a standardized data collection form. Any discrepancies between the authors were resolved through discussion to achieve consensus.

CBCT images that satisfied the inclusion criteria were analyzed and quantified by two periodontal experts (Feng Chen and Qi Liu), with the data recorded in a standardized table. In cases of disagreement, a third periodontal specialist was consulted. The inter-rater agreement was confirmed utilizing the Kappa test, ensuring the reliability of measurements.

**Figure 3:**
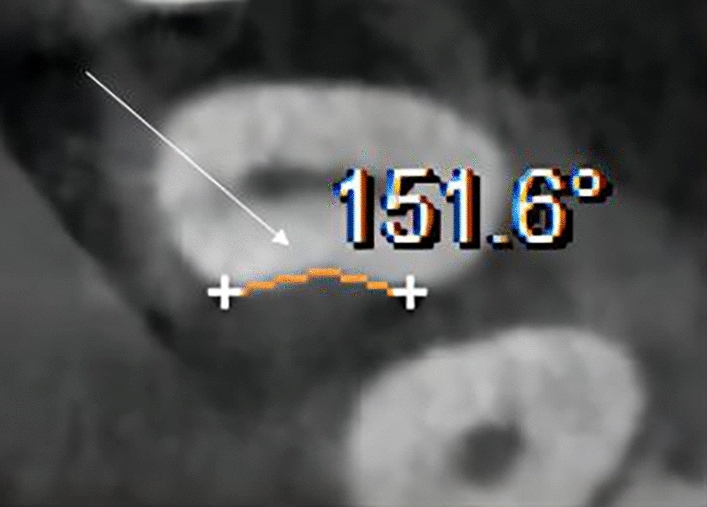
The angle of mesial concavity identified from the axial segment image.

The evaluation and measurement protocol for each CBCT image involved documenting the incidence and angle of mesial concavity in maxillary first premolars of patients with periodontitis. The measuring point for mesial concavity was at the enamel-cementum junction. The angle was determined by connecting the most convex points on the buccal and lingual sides with the most concave midpoint. The angle of mesial concavity ascertained from the axial image is depicted in Fig. 3.

**Figure 4:**
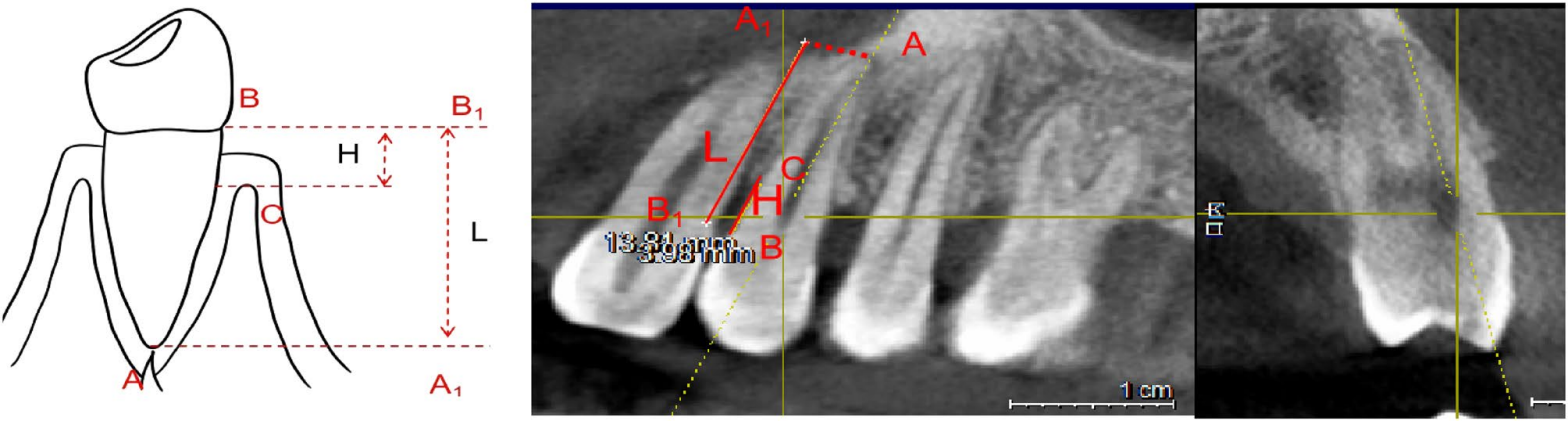
The apical point (**A**); cementum-enamel junction (CEJ) point (**B**); the alveolar ridge point (**C**); B1 was parallel to B; A1 was parallel to A; H was the line of B to C; L was the line of B1 to A1.

As demonstrated in Fig. 4, axial views at the enamel-cementum junction revealed the pulp chamber, while sagittal views intersecting its center and coronal views at either the buccal or palatal side provided insights into the alveolar bone condition. Reference points utilized were the apical point (A), the CEJ point (B), and the alveolar ridge point (C). A parallel line to point B (B1) and point A (A1) were projected to establish a line from B to A1, parallel to the coronal plane. Line L extended from B to C, and line H from B1 to A1. The extent of bone loss on the buccal or palatal side of the maxillary first premolar was calculated using the formula [(L − 2 mm)/(H − 2 mm)] × 100%^37^, indicating the percentage of bone loss.

### Statistical analyses

Statistical analysis was performed using the IBM SPSS Statistics software (version 24.0 for Windows). The association between tooth location and sex with the presence of mesial concavity in periodontitis patients was evaluated using the chi-squared test. The influence of mesial concavity on the gingival index, plaque index, and percentage of bleeding on probing in periodontitis patients was evaluated using the chi-squared test. The influence of mesial concavity on clinical attachment loss was examined using independent sample t-tests. The Mann–Whitney U test was applied to assess the impact of root concavity on the degree of bone loss and the severity of periodontitis in the maxillary first premolars with the degree of mesial concavity. The significance level was established at α = 0.05.

### Ethics approval and consent to participate

All study procedures involving human participants were in compliance with the ethical standards of the Declaration of Helsinki and its later amendments, as well as other relevant ethical guidelines. The research received ethical approval from the Ethics Committee of Hangzhou Normal University Hospital (Approval No. 2022(E2)-KS-107). Prior to enrollment in the study, participants were informed about the research objectives and procedures and provided written informed consent. Consent for the publication of identifiable images was obtained from all participants. Informed consent was acquired from each patient or their legal guardians for inclusion in the study.

## Data Availability

On reasonable request, the corresponding author will provide the datasets created and/or used in the current study.
